# Metabolomic profiling of *Burkholderia pseudomallei* using UHPLC-ESI-Q-TOF-MS reveals specific biomarkers including 4-methyl-5-thiazoleethanol and unique thiamine degradation pathway

**DOI:** 10.1186/s13578-015-0018-x

**Published:** 2015-06-02

**Authors:** Susanna K. P. Lau, Ching-Wan Lam, Shirly O. T. Curreem, Kim-Chung Lee, Wang-Ngai Chow, Candy C. Y. Lau, Siddharth Sridhar, Sally C. Y. Wong, Paolo Martelli, Suk-Wai Hui, Kwok-Yung Yuen, Patrick C. Y. Woo

**Affiliations:** State Key Laboratory of Emerging Infectious Diseases, Department of Microbiology, The University of Hong Kong, Room 423, University Pathology Building, Queen Mary Hospital, Pok Fu Lam, Hong Kong; Research Centre of Infection and Immunology, The University of Hong Kong, Pok Fu Lam, Hong Kong; Carol Yu Centre for Infection, The University of Hong Kong, Pok Fu Lam, Hong Kong; Department of Microbiology, The University of Hong Kong, Pok Fu Lam, Hong Kong; Department of Pathology, The University of Hong Kong, Pok Fu Lam, Hong Kong; Ocean Park Corporation, Aqua City, Hong Kong

**Keywords:** *Burkholderia pseudomallei*, Biomarkers, Specific, Metabolomics

## Abstract

**Background:**

*Burkholderia pseudomallei* is an emerging pathogen that causes melioidosis, a serious and potentially fatal disease which requires prolonged antibiotics to prevent relapse. However, diagnosis of melioidosis can be difficult, especially in culture-negative cases. While metabolomics represents an uprising tool for studying infectious diseases, there were no reports on its applications to *B. pseudomallei*. To search for potential specific biomarkers, we compared the metabolomics profiles of culture supernatants of *B. pseudomallei* (15 strains), *B. thailandensis* (3 strains), *B. cepacia* complex (14 strains), *P. aeruginosa* (4 strains) and *E. coli* (3 strains), using ultra-high performance liquid chromatography-electrospray ionization-quadruple time-of-flight mass spectrometry (UHPLC-ESI-Q-TOF-MS). Multi- and univariate analyses were used to identify specific metabolites in *B. pseudomallei*.

**Results:**

Principal component and partial-least squares discrimination analysis readily distinguished the metabolomes between *B. pseudomallei* and other bacterial species. Using multi-variate and univariate analysis, eight metabolites with significantly higher levels in *B. pseudomallei* were identified. Three of the eight metabolites were identified by MS/MS, while five metabolites were unidentified against database matching, suggesting that they may be potentially novel compounds. One metabolite, m/z 144.048, was identified as 4-methyl-5-thiazoleethanol, a degradation product of thiamine (vitamin B_1_), with molecular formula C_6_H_9_NOS by database searches and confirmed by MS/MS using commercially available authentic chemical standard. Two metabolites, m/z 512.282 and m/z 542.2921, were identified as tetrapeptides, Ile-His-Lys-Asp with molecular formula C_22_H_37_N_7_O_7_ and Pro-Arg-Arg-Asn with molecular formula C_21_H_39_N_11_O_6_, respectively. To investigate the high levels of 4-methyl-5-thiazoleethanol in *B. pseudomallei*, we compared the thiamine degradation pathways encoded in genomes of *B. pseudomallei* and *B. thailandensis*. While both *B. pseudomallei* and *B. thailandensis* possess thiaminase I which catalyzes degradation of thiamine to 4-methyl-5-thiazoleethanol, *thiM*, which encodes hydroxyethylthiazole kinase responsible for degradation of 4-methyl-5-thiazoleethanol, is present and expressed in *B. thailandensis* as detected by PCR/RT-PCR, but absent or not expressed in all *B. pseudomallei* strains. This suggests that the high 4-methyl-5-thiazoleethanol level in *B. pseudomallei* is likely due to the absence of hydroxyethylthiazole kinase and hence reduced downstream degradation.

**Conclusion:**

Eight novel biomarkers, including 4-methyl-5-thiazoleethanol and two tetrapeptides, were identified in the culture supernatant of *B. pseudomallei*.

**Electronic supplementary material:**

The online version of this article (doi:10.1186/s13578-015-0018-x) contains supplementary material, which is available to authorized users.

## Background

*Burkholderia pseudomallei* is an emerging, highly pathogenic, Gram-negative betaproteobacterium that causes melioidosis, a potentially serious and fatal disease often manifested as severe community-acquired pneumonia and sepsis. The bacterium is classified as a category B bioterrorism agent by the Center for Disease Control, USA. Although melioidosis is mainly endemic in Southeast Asia and northern Australia, the disease has been increasingly reported in countries outside the Asia-Pacific region, such as India [1, 2], Mauritius [3], South, Central and North America [4–6], and West and East Africa [7, 8], suggesting an expanding geographical distribution. *B. pseudomallei* is a natural saprophyte and melioidosis is believed to be acquired through environmental contact with contaminated soil and water [9, 10]. Illness can be presented as an acute, subacute, or chronic process, with an incubation period of up to 26 years [11]. The disease manifestations can range from subclinical infection, localized abscesses, pneumonia to fulminant sepsis, leading to a mortality rate of up to 19 % [12]. Besides human, melioidosis also affects a wide range of animals in endemic areas [10, 13]. Treatment of melioidosis is often difficult, as *B. pseudomallei* is usually resistant to multiple antibiotics and prolonged antibiotics are required to prevent relapse [14, 15]. Moreover, diagnostic and therapeutic resources in endemic areas are often limited, which have hindered efforts to improve treatment outcomes.

Diagnosis of melioidosis can be difficult, as the bacterium may not be readily isolated from clinical specimens. And even with positive cultures, commercial bacterial identifications often fail to differentiate between *B. pseudomallei* and closely related species such as *B. thailandensis* and members of *B. cepacia* complex [16]. Therefore, new molecular techniques are often required for more accurate species identification [14, 17–23]. Despite these new technologies, the diagnostic problems associated with culture-negative cases remain unresolved. Although different serological tests have been developed to help diagnose culture-negative melioidosis, their clinical usefulness is limited by the low sensitivities and specificities [24, 25]. Similarly, PCR assays for direct detection from blood and sputum samples are often associated with suboptimal sensitivities and specificities [23]. The availability of alternative techniques for improved diagnosis of melioidosis is thus eagerly awaited, and such techniques should be able to differentiate between melioidosis and infections caused by common Gram-negative bacteria including the closely related *Burkholderia* species.

Metabolomics is an uprising research platform for systematic studies of the small-molecular metabolite profiles of a biological system such as cell, tissue or organism, which may be intermediate of end products of various metabolic pathways. Using statistical analyses, the metabolomes of different biological systems can be compared, which may help differentiate between them and identify potential metabolite markers specific to each system. The technique has also been recently applied to characterize various infectious diseases and pathogens, with an aim to improve laboratory diagnosis [26–31]. In addition, the application of metabolomics to pathogens has opened a new arena for studying microbial metabolomics pathways while making use of genomic data. For example, we have recently reported a novel biosynthetic pathway for monascorubin, a thousand-year-old natural food colorant, in the pathogenic dimorphic fungus, *Penicillium marneffei*, identified using metabolomics approach and genomics data [32]. Despite being an important pathogen, no studies have reported the use of metabolomics to explore specific biomarkers in *B. pseudomallei*. We hypothesize that there are potentially novel extracellular metabolites specifically produced by *B. pseudomallei* that may be detected in body fluids of patients with melioidosis. To search for potential biomarkers for diagnosis of melioidosis, we attempted to characterize the metabolomes of culture supernatants of *B. pseudomallei* and related species, using ultra-high performance liquid chromatography-electrospray ionization-quadruple time-of-flight mass spectrometry (UHPLC-ESI-Q-TOF-MS). Multi-and univariate statistical analyses of the metabolome data were used to identify specific metabolites in *B. pseudomallei*.

## Results

### Visual inspection of total ion chromatograms

**Fig. 1:**
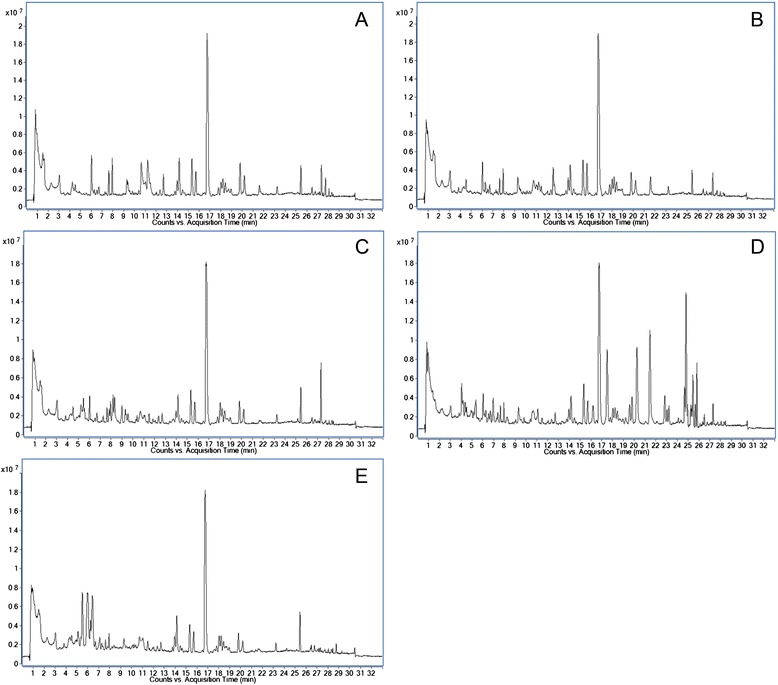
Total ion chromatogram of bacterial culture supernatant. Total ion chromatogram of culture supernatant of (**a**) *B. pseudomallei*, (**b**) *B. thailandensis*, (**c**) *B. cepacia* complex, (**d**) *P. aeruginosa* and (**e**) *E. coli* with UPLC-QTOF-MS performed under ESI positive mode. A representative chromatogram for each sample group was shown

We characterized and compared the metabolomes of culture supernatants from 15 *B. pseudomallei* (seven clinical and eight environmental isolates), three *B. thailandensis*, 14 *B. cepacia* complex, four *P. aeruginosa* and three *E. coli* isolates. The total ion chromatograms from the same bacterial species shared considerable similarity, whereas significant differences were observed in the chromatograms obtained between different species, except *B. pseudomallei* and *B. thailandensis* sharing higher similarity. Representative examples of chromatograms obtained from each species are shown in Fig. 1.

### PCA and PLS-DA modeling

**Fig. 2:**
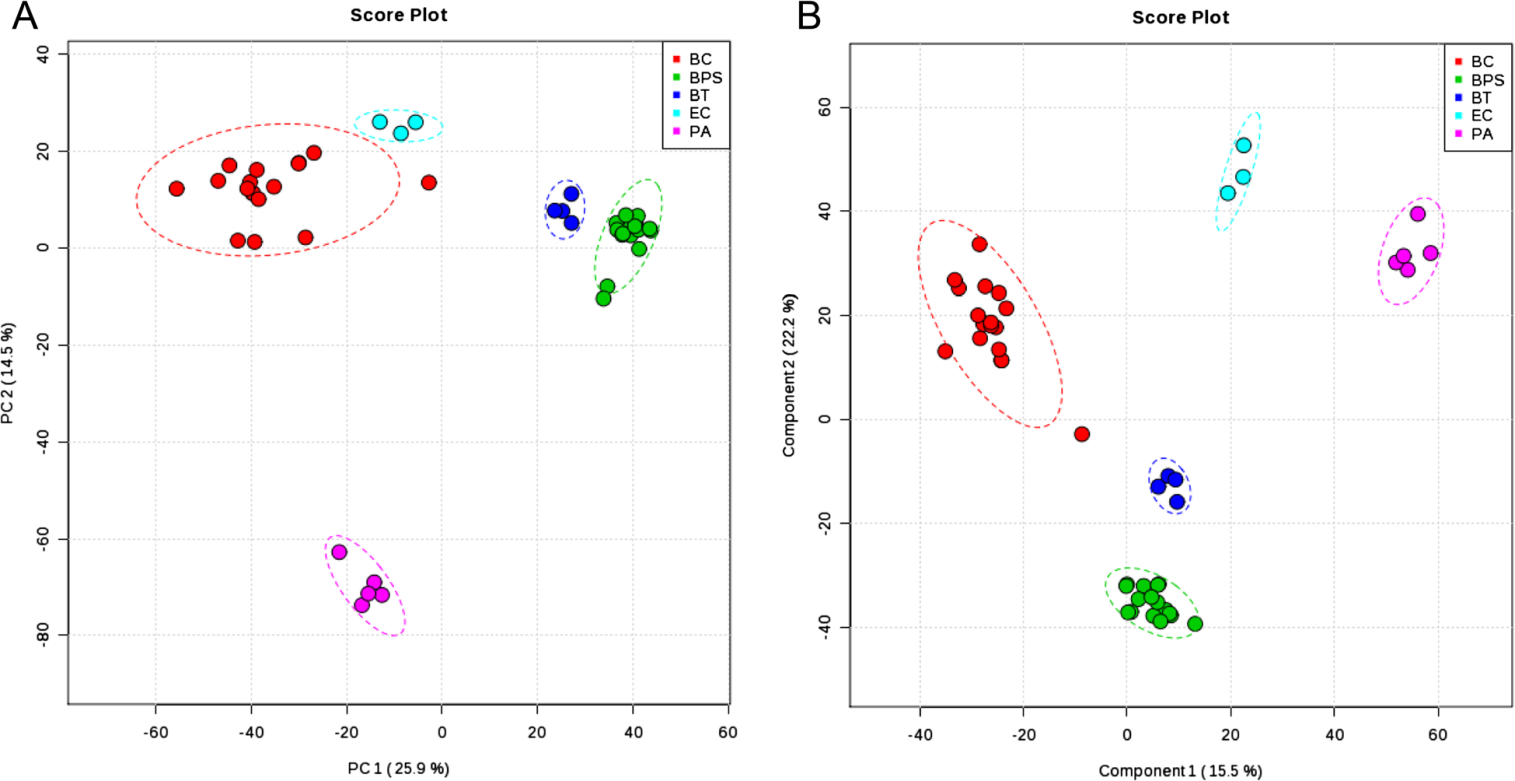
PCA and PLS-DA score plot based on culture supernatant. (**a**) PCA score plot and (**b**) PLS-DA score plot generated using MetaboAnalyst based on culture supernatant in positive mode. PLS-DA models were validated using *R* ^*2*^ and *Q* ^2^ based on leave one out cross-validation (LOOCV). Five-component model was selected as optimized model with *R* ^*2*^ = 0.99 and Q^2^ = 0.99. The significance of the model was demonstrated by permutation test with 2000 testing iterations using separation distance and *P* value <0.001 was obtained. BC, *B. cepacia* complex; BPS, *B. pseudomallei*; BT, *B. thailandensis*; PA, *P. aeruginosa*; EC, *E. coli*

**Fig. 3:**
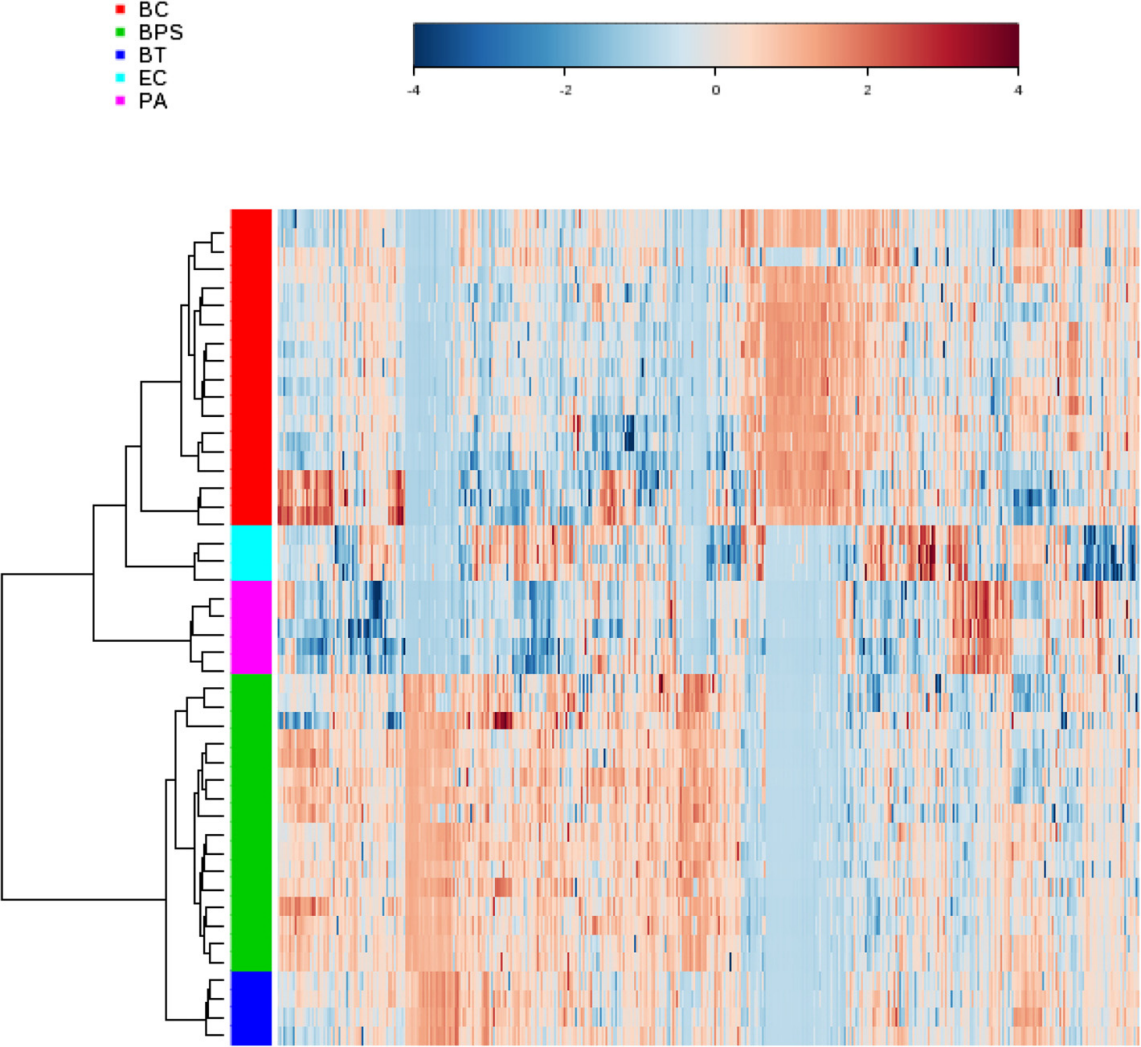
Hierarchical clustering analysis based on culture supernatant. Hierarchical clustering analysis generated using MetaboAnalyst based on culture supernatant in positive mode. Each bar represented a metabolite colored by its abundance intensities on normalized scale from blue (decreased level) to red (increased level). The dendrogram on the left was constructed based on the metabolite abundance profiles. BC, *B. cepacia* complex; BPS, *B. pseudomallei*; BT, *B. thailandensis*; PA, *P. aeruginosa*; EC, *E. coli*

To compare the metabolomes between *B. pseudomallei* and other bacterial species, both multi- and univariate analyses were performed. For multi-variate analysis, principal component analysis (PCA) showed that 40.4 % of the total variance in the data was represented by the first two principal components (PCs) (Fig. 2a). The 2D-PCA score plot revealed that all bacterial strains of the same species were closely related and can be distinguished from other bacterial species based on the first two principal components, with the *B. pseudomallei* strains clearly separated from other species including *B. thailandensis* along PC1 which represented 25.9 % of the variance. In view of the significant separation achieved using PCA, supervised partial-least squares discrimination analysis (PLS-DA) (Fig. 2b) was subsequently performed to maximize the separation and identify additional metabolites to those identified using PCA. In the PLS-DA score plot, the separation between different bacterial species is more prominent. Potential metabolites were selected based on the VIP score (>1). Based on the degree of similarity of metabolite abundance profiles, hierarchical clustering analysis was performed to show the global overview of all culture supernatant metabolites detected (Fig. 3). Metabolites with similar abundance pattern were positioned closer together. The heat map and dendrogram indicated the close clustering of *B. pseudomallei* strains and their separation from other bacterial species, although *B. pseudomallei* strains were more closely related to *B. thailandensis* strains than to other species. However, no significant difference was observed between clinical and environmental strains of *B. pseudomallei*. To further confirm the specificity and significance of potential metabolites identified from PCA and PLS-DA, univariate analysis of each metabolite was performed using one-way ANOVA and Student’s *t*-test. A total of eight potential metabolites contributing most to the variation between *B. pseudomallei* and other bacterial species with significantly higher level in *B. pseudomallei* strains were selected for further identification (Table 1).

**Table 1:** Specific metabolites in culture supernatant of *B. pseudomallei*

	m/z	Retention time (min)	Ionization mode	Ion	MS/MS Fragment masses	*P* value^a^	VIP score^b^	Molecular formula	Putative identity
1	468.3039	4.01	Positive	[M + H]^+^	86.0983, 98.0997, 125.1071, 143.0960, 198.1260, 266.1878, 281.1615, 366.2501, 392.2288, 425.2962, 451.2754, 468.3016	<0.001	2.15	C_19_H_41_N_5_O_8_	No match
2	144.048	4.28	Positive	[M + H]^+^	70.9969, 80.0513, 85.0126, 112.0226, 113.0289, 126.0414	<0.001	1.98	C_6_H_9_NOS	4-methyl-5-thiazoleethanol^c^
3	433.2228	5.78	Positive	[M + H]^+^	72.0810, 116.0699, 142.0961, 158.0929, 175.1184, 360.1502, 415.2908	<0.001	1.94	C_17_H_32_N_6_O_5_S	No match
4	512.282	7.16	Positive	[M + H]^+^	110.0709, 195.0869, 261.0912, 283.0697, 294.1568, 512.2825	<0.001	1.74	C_22_H_37_N_7_O_7_	Ile His Lys Asp
5	542.2921	7.89	Positive	[M + H]^+^	70.0654, 86.0963, 90.9760, 183.1480, 274.1853, 283.0696, 421.2550	<0.001	2.12	C_21_H_39_N_11_O_6_	Pro Arg Arg Asn
6	580.2922	10.1	Positive	[M + H]^+^	72.081, 112.0866, 175.1187, 305.1612, 433.2229	<0.001	2.05	C_21_H_41_N_9_O_8_S	No match
7^d^	571.3469/547.3464	21.97	Positive/Negative	[M + Na]^+^/[M-H]^−^	193.0939, 264.1313, 329.2208, 355.2353, 373.2456, 509.3097, 527.3206,553.3355, 571.3465/ 110.0236, 240.1339, 253.1662, 279.2084, 297.1562, 459.3365, 503.3224, 547.3513	<0.001/<0.001	2.16/ 2.17	C_29_H_48_N_4_O_6_	No match
8	763.5541	22.28	Positive	[M + H]^+^	90.9760, 125.1068, 143.0912, 158.9620, 226.9500, 288.9223, 578.4567, 661.4984, 703.5211, 720.5478, 746.5287, 764.5065	<0.001	2.17	C_38_H_70_N_10_O_6_	No match

^a^ *p* value from ANOVA analysis

^b^VIP score based on PLS-DA. VIP score >1 is considered to be statistically significant

^c^Confirmed by MS/MS fragmentation pattern matching with commercially available authentic chemical standard

^d^Detected in both positive and negative mode

### Identification of potential biomarkers specific to *B. pseudomallei*

The metabolites were identified by MS/MS fragmentation and their predicted molecular formulae were shown in Table 1. All metabolites except m/z 144.048 were found only in *B. pseudomallei* strains but not other bacterial species. Three (m/z 144.048, m/z 512.282 and m/z 542.2921 m/z 144.048) of the eight metabolites were identified as known compounds, while the other five metabolites may represent potentially novel metabolites with no match against known compounds or databases.

**Fig. 4:**
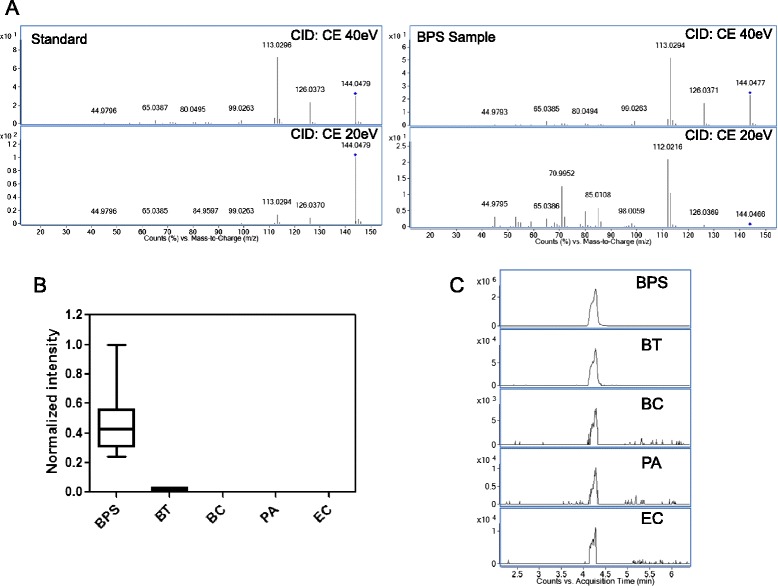
MS/MS mass spectra, box-whisker plots, extracted ion chromatogram and of m/z 144.041 and 4-methyl-5-thiazoleethanol standard. (**a**) MS/MS mass spectra, (**b**) box-whisker plots and (**c**) extracted ion chromatogram of m/z 144.041 and 4-methyl-5-thiazoleethanol standard. MS/MS fragmentations were performed at 10, 20 and 40 eV. BC, *B. cepacia* complex; BPS, *B. pseudomallei*; BT, *B. thailandensis*; PA, *P. aeruginosa*; EC, *E. coli*

The metabolite m/z 144.048 was identified as 4-methyl-5-thiazoleethanol (metabolite no. 2 in Table 1) with molecular formula C_6_H_9_NOS by database searches in METLIN and Massbank, and confirmed by MS/MS using commercially available authentic chemical standard of 4-methyl-5-thiazoleethanol (Fig. 4a). 4-methyl-5-thiazoleethanol is a degradation product of thiamine (vitamin B_1_), an essential cofactor in most living organisms, being important for purine metabolism. Although it was found in *B. pseudomallei* strains with significantly higher levels (Fig. 4b), low levels of m/z 144.048 (at approximately 100-to 1000-fold lower levels) were also detected in *B. thailandensis*, *B. cepacia* complex, *P. aeruginosa* and *E. coli* (Fig. 4c).

Two metabolites, m/z 512.282 and m/z 542.2921 were identified as tetrapeptides (metabolite no. 4 and 5 in Table 1). m/z 512.282 with molecular formula C_22_H_37_N_7_O_7_ was identified as Ile-His-Lys-Asp, and m/z 542.2921 with molecular formula C_21_H_39_N_11_O_6_ was identified as Pro-Arg-Arg-Asn in METLIN. Some bacteria are known to produce short peptides as pheromones, which are involved in quorum-sensing [33]. Future studies are required to determine the origin and biological significance of these tetrapeptides in *B. pseudomallei*.

### Phylogenetic analysis of thiaminase I and hydroxyethyl thiazole kinase genes of *B. pseudomallei* and *B. thailandensis*

**Fig. 5:**
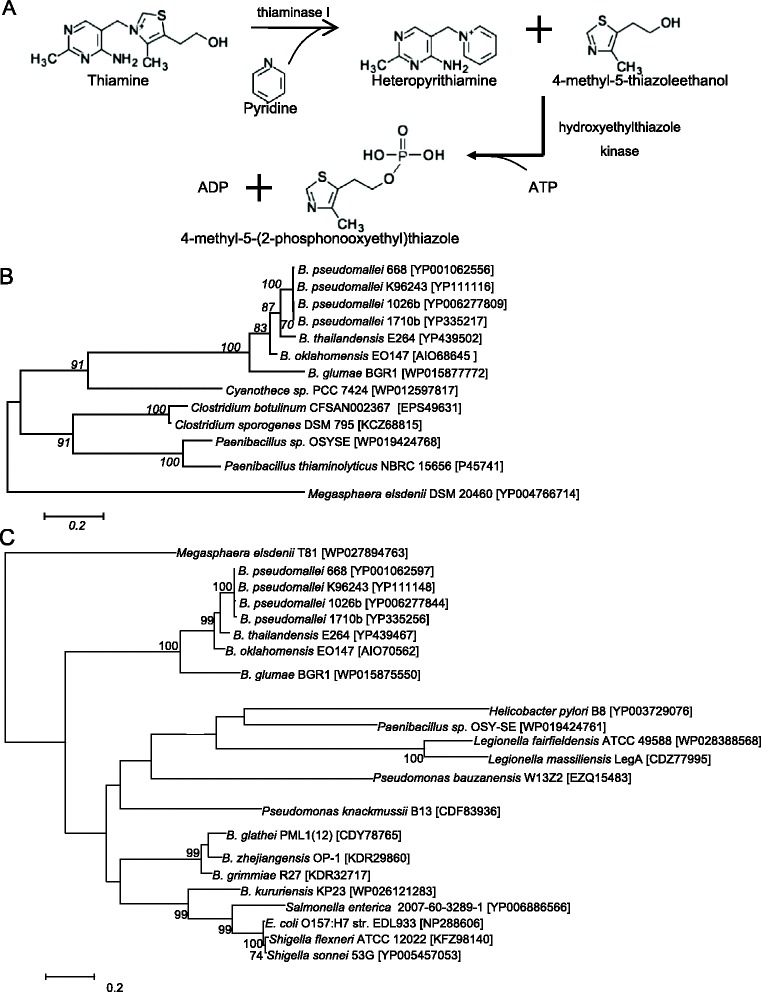
Function and phylogeny of thiaminase I and hydroxyethylthiazole kinase. **a** Thiaminase I (EC 2.5.1.2) catalyzes the degradation of thiamin by replacing the thiazole moiety with a variety of nucleophiles. Hydroxyethylthiazole kinase (EC 2.7.1.50) catalyzes the degradation of 4-methyl-5-thiazoleethanol by transferring the phosphorus-containing groups with an alcohol group as acceptor with ATP. **b** Phylogenetic tree showing the relationship of Thiaminase I in *B. pseudomallei* and *B. thailandensis* to homologues in other bacteria constructed by maximum-likelihood method. A total 341 amino acid positions were included in the analysis. Bootstrap values were calculated as percentages from 1000 replicates and only values ≥70 % were shown. The scale bars indicate the estimated number of substitutions per 5 amino acids. Names and accession numbers are given as cited in GenBank database. **c** Phylogenetic tree showing the relationship of hydroxyethylthiazole kinase in *B. pseudomallei* and *B. thailandensis* to homologues in other bacteria constructed by maximum-likelihood method. A total 261 amino acid positions were included in the analysis. Bootstrap values were calculated as percentages from 1000 replicates and only values ≥70 % were shown. The scale bars indicate the estimated number of substitutions per 5 amino acids. Names and accession numbers are given as cited in GenBank database

To investigate the high levels of 4-methyl-5-thiazoleethanol in *B. pseudomallei* culture supernatant, we attempted to compare the thiamine degradation pathways in *B. pseudomallei* and *B. thailandensis*. The thiamine degrading enzyme, thiaminase I, which catalyzes the degradation of thiamine to 4-methyl-5-thiazoleethanol (Fig. 5a), can be found in available genome sequences of *B. pseudomallei* and *B. thailandensis* [34]. Phylogenetic analysis of all bacterial thiaminase I genes available from GenBank showed that the sequences from *B. pseudomallei* are most closely related to that from *B. thailandensis* (Fig. 5b). Moreover, the thaiminase I genes of *B. pseudomallei* and *B. thailandensis* were clustered with those of two other *Burkholderia* species with sequences available, forming a distinct cluster among all bacterial sequences. Other known bacterial thiaminase I genes have only been found in phylogenetically distant bacterial species, such as those Gram-positive bacteria like *Clostridium, Paenibacillus* and *Bacillus*. The findings suggested that *Burkholderia* species is unique among Gram-negative bacteria in having acquired this gene which may be involved in specific functions. Since both *B. pseudomallei* and *B. thailandensis* possessed a thiaminase I homologue, the higher levels of 4-methyl-5-thiazoleethanol in *B. pseudomallei* are unlikely to be related to thiaminase I.

On the other hand, hydroxyethylthiazole kinase, encoded by the gene, *thiM*, catalyzes the degradation of 4-methyl-5-thiazoleethanol. Unlike thiaminase I gene, *thiM* is widely distributed in various Gram-negative bacteria. The gene can also be found in available genome sequences of *B. pseudomallei* and *B. thailandensis* [34]. Phylogenetic analysis showed that the sequences from *B. pseudomallei* are most closely related to those from *B. thailandensis* and other *Burkholderia* species (Fig. 5c).

### PCR for thiaminase I and hydroxyethylthiazole kinase gene and RT-PCR for mRNA detection in *B. pseudomallei* and *B. thailandensis*

**Fig. 6:**
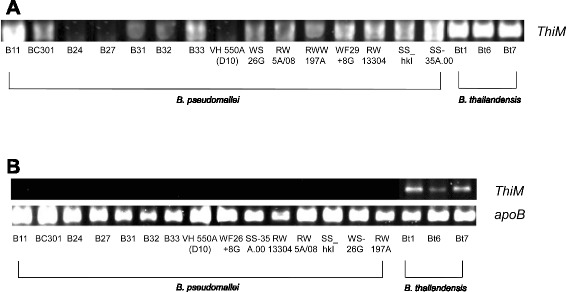
PCR for hydroxyethyl thiazole kinase genes and RT-PCR for mRNA detection in *B. pseudomallei* and *B. thailandensis.* **a** PCR of *thiM* from genomic DNA: *thiM* is present in the genomes of the three *B. thailandensis* strains, Bt1, Bt6 and Bt7, and 12 of 15 *B. pseudomallei* strains, but absent in the genomes of three *B. pseudomallei* strains, B24, B27 and VG550A (D10) with the highest 4-methyl-5-thiazoleethanol levels. **b** RT-PCR of *thiM* from mRNA of *B. thailandensis*: *thiM* is expressed in *B. thailandensis* strains Bt1, Bt6 and Bt7, but not expressed in the 12 *B. pseudomallei* strains that possessed *thiM* gene. Housekeeping gene *apoB* is used for normalization

PCR and RT-PCR using specific primers targeting thiaminase I gene showed that it is present and expressed in all the 15 *B. pseudomallei* and three *thailandensis* strains. Therefore, it is unlikely that the increased 4-methyl-5-thiazoleethanol level in *B. pseudomallei* is due to thiaminase I regulation. On the other hand, to test whether the higher levels of 4-methyl-5-thiazoleethanol in *B. pseudomallei* may be related to reduced downstream degradation, we attempted to detect the *thiM* gene, encoding the enzyme, hydroxyethylthiazole kinase, responsible for downstream degradation of 4-methyl-5-thiazoleethanol to 4-methyl-5-(2-phosphonooxyethyl)thiazole. PCR using specific primers targeting *thiM* gene showed that *thiM* gene is present in the three *B. thailandensis* strains and 12 of 15 *B. pseudomallei* strains, while it is absent in the three *B. pseudomallei* strains (B24, B27, VG550A(D10)) showing the highest 4-methyl-5-thiazoleethanol levels (Fig. 6a). To detect the mRNA expression of *thiM*, RT-PCR using specific primers was performed. The results showed that the *thiM* gene is expressed in all the three *B. thailandensis* strains but not expressed in all 12 *B. pseudomallei* strains that possessed the *thiM* gene (Fig. 6b).

## Discussion

Using metabolomics approach, we identified specific metabolites in culture supernatant of *B. pseudomallei*. As these extracellular metabolites are either secreted or released from cell wall components of *B. pseudomallei*, they may be present in the circulating blood or other body fluids of infected patients, and hence may represent potential biomarkers for diagnosis. The exclusion of metabolites present in Gram-negative bacteria such as *Enterobacteriaceae* and other non-fermenters was important, since these bacteria are common causes of bacteremia. Moreover, differentiation of *B. pseudomallei* from *B. thailandensis* in clinical isolates may be important for accurate diagnosis of melioidosis, since *B. thailandensis* is much less virulent and was only rarely reported to cause invasive infections in humans [10, 35]. Therefore, the identification of specific biomarkers for *B. pseudomallei* may help differentiate melioidosis from other Gram-negative bacterial infections. In this study, both total ion chromatograms and statistical analyses showed that the metabolomes of the culture supernatant of *B. pseudomallei* strains are significantly different from those of other tested Gram-negative species including *B. thailandensis* and *B. cepacia* complex. Using both mutli-and uni-variate analyses, eight metabolites with significantly higher levels in *B. pseudomallei* were identified and subject to MS/MS fragmentation for identification. Most of the metabolites were unidentified by MS/MS against database matching, suggesting that these are potential novel compounds. This is not unexpected, since metabolomics for the study of microbes is still an emerging field and the number of known metabolites from *B. pseudomallei* is very limited. Further studies on the chemical structure and biosynthetic pathway of these potential novel metabolites would help understand their biological function in *B. pseudomallei*. More importantly, metabolomics studies on clinical samples from patients with melioidsosis are warranted to determine if these *B. pseudomallei*-specific extracellular metabolites are present in sufficient quantities in clinical samples of infected patients to serve as diagnostic biomarkers for melioidosis.

The presence of high levels of 4-methyl-5-thiazoleethanol in the culture supernatant of *B. pseudomallei* strains is intriguing. Currently, two classes of thiamine degrading enzymes, thiaminase I and II, have been identified. Thiaminase I (EC 2.5.1.2) catalyzes the reaction involving the replacement of organic nucleophiles with the thiazole group in thiamine. The enzyme can be found in shellfish, fish and plants, but is only reported in a limited number of bacteria such as *Bacillus* and *Clostridium* [36]. Thiaminase II (EC 3.5.99.2), although initially identified with thiamine degrading activity, is subsequently revealed to be involved in salvage pathway of thiamine pyrimidine from base-degraded thiamine [37, 38]. *TenA*, the gene that encodes thiaminase II, can be found in bacteria, archaea and eukaryotes [38]. In *B. pseudomallei*, a homologue of the gene encoding thiaminase I, but not *TenA*, can be found in the available complete genome sequences of *B. pseudomallei* [39]. Therefore, 4-methyl-5-thiazoleethanol detected in *B. pseudomallei* is most likely a product of thiaminase I. The homologue of thiaminase I in *B. pseudomallei* is most closely related to that of *B. thailandensis*, *B. oklahomensis* and *B. glumae*. Interestingly, such homologue is absent in other common Gram-negative bacteria such as *P. aeruginosa* and *E. coli*, supporting a unique function among some *Burkholderia* species. Nevertheless, low levels of 4-methyl-5-thiazoleethanol (at 100-to 1000-fold lower levels) were also detected in *B. cepacia* complex, *P. aeruginosa* and *E. coli* in this study, which may be due to the presence of other proteins sharing conserved domains with thiaminase [40].

Since both *B. pseudomallei* and *B. thailandensis* possessed a thiaminase I homologue with mRNA expression, we hypothesize that the higher levels of 4-methyl-5-thiazoleethanol in *B. pseudomallei* than in *B. thailandensis* may be due to accumulation of this compound as a result of reduced downstream degradation. Hydroxyethylthiazole kinase (EC 2.7.1.50) is the enzyme responsible for degradation of 4-methyl-5-thiazoleethanol. It is a phosphotransferase that catalyzes the transfer of a phosphorus-containing group of ATP with the alcohol group of 4-methyl-5-thiazoleethanol as the acceptor, resulting in ADP and 4-methyl-5-(2-phosphonooxyethyl)thiazole (Fig. 5a). Although *thiM* gene that encodes hydroxyethylthiazole kinase can be found in the available genomes of *B. pseudomallei* [34], a previous transcriptome study on *B. pseudomallei* strain K94263 showed no detectable expression of *thiM* when the bacterium was cultured under 82 different conditions [41]. In contrast, a transcriptome study on *B. thailandensis* type strain E264, *thiM* was found to be expressed [42]. Furthermore, *thiM* has been classified as one of the non-core genes in *B. pseudomallei* and was found to be variably present among 94 studied strains in an array-based comparative genomic hybridization study [41, 43, 44]. Interestingly, in a recent report on whole genome analysis of two *B. pseudomallei* strains isolated 139 months apart from the same patient, *thiM* was among the 221 genes lost during reduction evolution [45]. We therefore tested our *B. pseudomallei* and *B. thailandensis* strains for the presence of *thiM* gene and mRNA expression. Our results showed that *thiM* gene is either absent or not expressed in all 15 *B. pseudomallei* strains showing high levels of 4-methyl-5-thiazoleethanol in culture supernatants. On the contrary, *thiM* gene is present and expressed in the three *B. thailandensis* strains showing low levels of 4-methyl-5-thiazoleethanol. Based on these data, it is likely that the higher level of 4-methyl-5-thiazoleethanol in *B. pseudomallei* than in *B. thailandensis* is the result of lack of *thiM* or hydroxyethylthiazole kinase activity.

The present study revealed a unique metabolic pathway of thiamine degradation in *B. pseudomallei* resulting in the accumulation of 4-methyl-5-thiazoleethanol likely as an end product. *Burkholderia* species are the only Gram-negative bacteria that possess thiaminase I gene for degradation of thiamine to 4-methyl-5-thiazoleethanol. Although *thiM* can be found in other Gram-negative bacteria, they cannot produce 4-methyl-5-thiazoleethanol from thiamine, which explains the very low levels observed in *E. coli* and *P. aeruginosa* strains. Among the genus *Burkholderia*, both thiaminase I and *thiM* genes can be found in available genome sequences of *B. pseudomallei*, *B. thailandensis, B. oklahomensis* and *B. glumae* in GenBank (Fig. 5b and c). The presence of *thiM* gene with mRNA expression in the three *B. thailandensis* strains, in line with the previous transcriptome study [42], supports hydroxyethylthiazole kinase activity and explains the low 4-methyl-5-thiazoleethanol level as a result of degradation to 4-methyl-5-(2-phosphonooxyethyl)thiazole. In contrast, although *thiM* gene can be found in some reported *B. pseudomallei* strains, previous studies showed that the gene may be absent, not expressed or lost during evolution [41, 43–45]. As supported by the present study, *B. pseudomallei* likely lacks hydroxyethylthiazole kinase activity and hence is unable to degrade 4-methyl-5-thiazoleethanol. Although *B. oklahomensis* and *B. glumae* also possess both thiaminase I and *thiM* genes, they are not pathogenic to humans. Therefore, 4-methyl-5-thiazoleethanol may represent a specific biomarker for clinical isolates suspicious of *B. pseudomallei*. Further studies may be performed to elucidate potential biological and pathogenic role of this unique metabolic pathway and high levels of 4-methyl-5-thiazoleethanol in *B. pseudomallei*.

Metabolomics is an emerging tool in studying microbes and infectious diseases. The technique offers a revolutionary approach to study both the pathogen itself as well as the host response to infections, providing insights on diagnosis, pathogenesis, host-pathogen interactions and metabolic pathways. Metabolomic data obtained from urine samples have been used to distinguish healthy subjects from patients with pneumococcal disease and urinary tract infections [46–48]. A study using nuclear magnetic resonance spectroscopy-based metabolomics also showed that the metabolic profile of sera from tuberculosis patients can be distinguished from those from healthy controls [49]. In another study using serum metabolomics approach on leprosy patients, higher levels of polyunsaturated fatty acids were found among patients having higher bacterial indices, which may provide clues on biological pathways involved in the immunomodulation of leprosy [50]. With the increasing applications of metabolomics technology on both microbial and clinical samples from patients with appropriate controls, we expect to witness an exponential expansion of our knowledge on microbial metabolites, including the discovery of novel metabolites and potential biomarkers for diagnosis of infections such as melioidosis. In particular, application of metabolomics for diagnosis of culture-negative melioidosis is potentially advantageous over other diagnostic methods such as serological and PCR assays. First, the detection of metabolites by mass spectrometry offers high specificity, devoid of the problem of cross-reacting antibodies as in serological assays and false-positive PCR reactions as a result of DNA from host or other bacteria. Second, the same metabolomics platform can be extended for detection of different metabolites useful for melioidosis and other infectious diseases. Although the equipment and expertise for metabolomics technology is currently not available in most clinical microbiology laboratories, the success of matrix-assisted laser-desorption ionization time-of-flight mass spectrometry as a revolutionary method for pathogen identification suggests that mass spectrometry may emerge as a new tool for diagnostic microbiology. New diagnostic tests for potential biomarkers may also be developed in other platforms that are easy to use in clinical laboratories.

## Conclusion

The present study illustrates the power of the state-of-the-art metabolomics technology in exploring potential biomarkers in *B. pseudomallei*. Eight metabolites with significant higher levels were identified in culture supernatants of *B. pseudomallei* compared to other tested bacterial species, including 4-methyl-5-thiazoleethanol and two tetrapeptides, Ile-His-Lys-Asp and Pro-Arg-Arg-Asn. The high level of 4-methyl-5-thiazoleethanol in *B. pseudomallei* is likely due to the absence of hydroxyethylthiazole kinase and hence reduced downstream degradation. Further studies of the *B. pseudomallei*-specific metabolites would help understand their biological significance and potential role as diagnostic biomarkers.

## Materials and methods

### Bacterial strains and culture

Fifteen *B. pseudomallei* (seven clinical and eight environmental isolates), three *B. thailandensis*, 14 *B. cepacia* complex, four *Pseudomonas aeruginosa* and three *Escherichia coli* isolates were included in this study (Additional file 1: Table S1). All isolates were phenotypically identified by the API 20NE system (bioMérieux Vitek, Hazelwood, MO), supplemented by conventional biochemical methods [51]. All *B. pseudomallei*, *B. thailandensis* and *B. cepacia* complex strains were isolated and characterized in previous studies [23, 52]. Each bacterial strain was grown on sheep blood agar at 37 °C overnight. Single colony was inoculated from blood agar to 5 ml RPMI 1640 medium (Gibco, Carlsbad, CA, USA) for incubation at 37 °C with shaking at 200 rpm for 24 h. The primary sub-cultures were adjusted to OD_600_ 0.2 and 1 mL of the diluted culture was further sub-cultured in 30 ml of fresh RPMI medium at 37 °C with shaking at 200 rpm for 24 h. The secondary sub-cultures were centrifuged at 3,000 rpm for 30 min to obtain the supernatant which was filtered twice using 0.22 μm filters. Metabolic activities in the filtrates were quenched immediately by incubating the filtrates in liquid nitrogen for 10 min. The filtrates were lyophilized and stored at–80 °C until sample extraction and analysis. Uninoculated culture medium was used as negative control.

### Chemicals and reagents

LC-MS grade water, methanol and acetonitrile was purchased from J.T. Baker (Center Valley, PA, USA). Analytical grade acetic acid, 5 M ammonium acetate and standard chemical 4-methyl-5-thiazoleethanol were purchased from Sigma-Aldrich, Inc (Saint Louis, MO, USA).

### Sample preparation

Lyophilized samples were reconstituted by dissolving in 1 mL solvent mixture containing water/methanol/acetonitrile (1:2:2). The samples were vortexed for 1 min and subsequently sonicated for 10 min at room temperature. After centrifugation at 15,000 × g for 15 min at 4 °C, supernatants were transferred to LC vial for LC-MS analysis.

### LC-MS system

For liquid chromatography, the separation was performed by Agilent 1290 UHPLC (Agilent Technologies, USA) and Agilent Eclipse Plus RRHD C18 (2.1 × 100 mm, 1.8 μm) column with Agilent Eclipse Plus RRHT C18 (2.1 × 30 mm, 1.8 μm) guard column. The injection volume was 3 μl. The column and autosampler temperature were maintained at 45 °C and 10 °C, respectively. The separation was performed at a flow rate of 0.4 mL/min under a gradient program in which mobile phase A was composed of 5 mM ammonium acetate in water containing 0.1 % acetic acid (v/v) and mobile phase B was composed of 0.05 % acetic acid (v/v) in acetonitrile. The gradient program was applied as follows: *t* = 0 min, 5 % B; *t* = 0.25 min, 5 % B; *t* = 20 min, 80 % B; *t* = 23 min, 99 % B; *t* = 33 min, 99.5 % B; *t* = 33.1 min, 99.5 % B. The stop time was 40 min. For MS, data was acquired by Agilent 6540 Q-TOF mass spectrometer (Agilent Technologies, USA) operating in the positive and negative ion mode using Agilent Jet Stream Electrospray ionization (ESI) source. The capillary voltage was set at +3800 V (positive mode) and-3800 V (negative mode) with nozzle voltages of +0 V and-0 V, respectively. Other source conditions were kept constant in all the experiments as follow: gas temperature was kept constant at 300 °C, drying gas (nitrogen) was set at the rate of 7 L/min, and the pressure of nebulizer gas (nitrogen) was 40 psi. The sheath gas was kept at a flow rate of 10 L/min and was maintained at a temperature of 330 °C. The voltages of the Fragmentor, Skimmer 1, and OctopoleRFPeak were 135 V, 65 V and 750 V respectively. The scan range was adjusted to 80–1700 m/z at the acquisition rate of 2 spectra/s. MS/MS acquisition was operated in the same parameter as in MS acquisition. Collision Energy (CE) was used at 20 or 40 eV for fragmentation of the targeted compounds.

### Data processing and statistical data analysis

All mass spectral data was acquired using Agilent MassHunter Qualitative Analysis software (version B.05.00, Agilent Technologies, USA). To optimize feature detection and discovery, two software packages: Mass Hunter Qualitative Analysis and open-source software XCMS (version 1.38.0) operating in R, which adopted different peak detection and alignment algorithms, were used [53]. For Mass Hunter Qualitative Analysis software, data preprocessing including baseline correction, noise calculations and molecular features extraction were performed with built-in small molecule extraction algorithm. Data was subsequently processed using Mass Profiler Professional (MPP) (Agilent Technologies) for peak alignment, data filtering and statistical analysis. For XCMS, raw data files were first converted to mzDATA format and peak detection were performed with centWave alogrithm in XCMS [54]. Data was subsequently processed using XCMS for peak alignment and data filtering. MetaboAnalyst 2.0 (www.metaboanalyst.ca) [55] was used for statistical analysis [55]. Further data processing including normalization, scaling and filtering were performed prior to statistical analysis in both software. Only variables that are present in at least 60 % of any group and with intensity of at least 4.0E + 03 were included for analysis in order to reduce noise and low abundance metabolites. The MS data were log_2_-transformed and mean-centered with unit variance scaling for statistical analysis. PCA and hierarchical clustering were performed for unsupervised multivariate statistical analysis. PLS-DA were performed as supervised method to identify important variables with discriminative power. PLS-DA models were validated based on multiple correlation coefficient (*R*^*2*^) and cross-validated R^2^ (*Q*^2^) in cross-validation and permutation test by applying 2000 iterations (*p* > 0.001). The significance of the biomarkers was ranked using the variable importance in projection (VIP) score (>1) from the PLS-DA model. For univariate analysis of candidate specific biomarkers in culture supernatant, statistical significance was determined using one-way ANOVA with Tukey’s *post*-*hoc* test between different bacterial species (*B. pseudomallei*, *B. thailandensis*, *B. cepacia* complex, *P. aeruginosa* and *E. coli*) and Student’s *t*-test for comparison between *B. pseudomallei* and other bacterial species. *P* < 0.05 was considered to be statistically significant. Volcano plot with fold change >5 and *P* < 0.05 was performed where appropriate. Box-whisker plots were produced using GraphPad Prism software (GraphPad Software Inc., California, USA). Extracted ion chromatograms of potential specific metabolites identified by statistical analysis were manually viewed to confirm the differences in peak areas between MTB and NTB samples. Metabolites were further filtered using CAMERA package in R, Mass Hunter and manual inspection to exclude possible fragments, dimers, adducts and isotopes [56]. Specific metabolites that were detected by both MPP and MetaboAnalyst to be statistically significant were considered to be potential biomarkers.

### Metabolite identification

MS/MS fragmentation was performed on the identified potential specific biomarkers. Identification of potential biomarkers was carried out by METLIN database (http://metlin.scripps.edu/) [57], Human Metabolome Database (HMDB) (http://www.hmdb.ca/) [58], *E. coli* Metabolome Database (ECMDB) (http://www.ecmdb.ca/) [59], MassBank (http://www.massbank.jp/) [60], LipidMaps (http://www.lipidmaps.org/) [61] and/or KEGG database (http://www.genome.jp/kegg) [62] search using exact molecular weights or MS/MS fragmentation pattern data and literature search. For confirmation of metabolite identity using authentic chemical standard, MS/MS fragmentation pattern of chemical standard was compared with that of candidate metabolite under same LC-MS condition to reveal any matching. In case of unknown metabolites, molecular formulae were generated using Mass Profiler Professional.

### Phylogenetic analysis of thiaminase I and hydroxyethyl thiazole kinase genes in *B. pseudomallei* and *B. thailandensis*

The protein sequences of bacterial thiaminase I and hydroxyethyl thiazole kinase were retrieved from Genbank. Phylogenetic trees were constructed using the maximum-likelihood method with 1000 bootstrap replicates with Mega 5.1 [63]. WAG + G amino acid substitution model with 5 gamma categories was used.

### PCR for thiaminase I and hydroxyethyl thiazole kinase genes and RT-PCR for mRNA detection in *B. pseudomallei* and *B. thailandensis*

Genomic DNA was extracted from 15 *B. pseudomallei* and three *B. thailandensis* strains using DNeasy mini Kit (Qiagen, Hilden, Germany). Total RNA was extracted using RNeasy mini Kit (Qiagen, Hilden, Germany) according to manufacturer’s instructions. The purified RNA was subjected to DNase I treatment using TURBO DNA-free kit (Ambion, USA). Reverse transcription was performed using the Superscript III kit (Invitrogen, Carlsbad, CA) according to manufacturer’s instructions. The resultant genomic DNA or cDNA s were then used for PCR amplification. The PCR mixture (25 μL) contained genomic DNA or cDNA (1.0 μL) as template, 0.5 μM primers, 2.5 μL 10× PCR buffer II, 2.5 mM MgCl_2_, 200 μM of each dNTPs (GeneAmp, Applied Biosystems, Waltham, Massachusetts, USA) and 1.0U Taq polymerase (AmpliTaq Gold; Applied Biosystems, Waltham, Massachusetts, USA). Thermal cycling was performed in an automated thermocycler (Veriti 96-well fast thermal cycler; Applied Biosystems, Waltham, Massachusetts, USA) with a hot-start at 95 °C for 10 min; 35 cycles of 95 °C for 30 s, annealing for 30 s at temperatures of 53 °C and 72 °C for 30 s and a final extension at 72 °C for 10 min. Five microliters of each amplified product was electrophoresed in 2.5 % (w/v) agarose gel with a molecular size marker (GeneRuler 50 bp DNA Ladder; Fermentas, Pittsburgh, PA, USA) in parallel. Electrophoresis in Tris-borate-EDTA buffer was performed at 120 V for 35 min. The gel was stained with ethidium bromide (0.5 μg/mL) for 25 min, rinsed and photographed under ultraviolet light illumination. Standard precautions were taken to avoid PCR contamination, and no false-positive was observed in negative controls. The primers used are as shown in Additional file 2: Table S2. Housekeeping gene *apoB* was used for normalization.

## Additional files

Additional file 1: Table S1. Bacterial strains used in this study. Additional file 2: Table S2. Primers used in this study.

## Abbreviations

UHPLC-ESI-Q-TOF-MS: Ultra high performance liquid chromatography-electrospray ionization-quadruple-time-of flight-mass spectrometry
MS/MS: Tandem mass spectrometry
LC-MS: Liquid chromatography-mass spectrometry
PCA: Principal component analysis
PLS-DA: Partial least squares Discriminant Analysis
