# Supplementary material for: Metabolomic profiling of Burkholderia pseudomallei using UHPLC-ESI-Q-TOF-MS reveals specific biomarkers including 4-methyl-5-thiazoleethanol and unique thiamine degradation pathway
Source: Cell Biosci. 2015 Jun 2;5:26. doi: 10.1186/s13578-015-0018-x (PMC4475313; doi:10.1186/s13578-015-0018-x)
Supplement: Additional file 1: Table S1. — Bacterial strains used in this study. [file 13578_2015_18_MOESM1_ESM.doc]

**Additional file 1: Table S1** **Bacterial strains used in this study**

| Species | strain | Source | Origin/Reference |
| --- | --- | --- | --- |
| *Burkholderia pseudomallei* | B11 | Patient isolate | This study |
|  | B24 | Patient isolate | This study |
|  | B27 | Patient isolate | This study |
|  | B31 | Patient isolate | This study |
|  | B32 | Patient isolate | This study |
|  | B33 | Patient isolate | This study |
|  | BC301 | Patient isolate | This study |
|  | RW13304 | Rainwater from an oceanarium | This study |
|  | RW5A/08 | Rainwater from an oceanarium | This study |
|  | RWW197A | Rainwater from an oceanarium | This study |
|  | SS_hkI | Soil from an oceanarium | This study |
|  | SS-35A.00 | Soil from an oceanarium | This study |
|  | VH550A(D10) | Soil from an oceanarium | This study |
|  | WF29+8G | Soil from an oceanarium | This study |
|  | WS-26G | Streamwater from an oceanarium | This study |
|  |  |  |  |
| *Burkholderia thailandensis* | Bt1 | Patient isolate | This study |
|  | Bt6 | Patient isolate | This study |
|  | Bt7 | Patient isolate | This study |
|  |  |  |  |
| *Burkholderia cepacia* complex |  |  |  |
| *Burkholderia anthina* | LMG20980^T^ | Soil | [1] |
| *Burkholderia arboris* | BCEP5 | Patient isolate | [2] |
| *Burkholderia arboris* | BCEP13 | Patient isolate | [2] |
| *Burkholderia cepacia* | BCEP10 | Patient isolate | [2] |
| *Burkholderia cepacia* | BCEP11 | Patient isolate | [2] |
| *Burkholderia cepacia* | BCEP12 | Patient isolate | [2] |
| *Burkholderia cepacia* | LMG1222^T^ | Onion (*Allium cepa*) | [3] |
| *Burkholderia cenocepacia* | BCEP15 | Patient isolate | [2] |
| *Burkholderia cenocepacia* | BCEP16 | Patient isolate | [2] |
| *Burkholderia cepacia* complex | BCEP3 | Patient isolate | [2] |
| *Burkholderia contaminas* | BCEP17 | Patient isolate | [2] |
| *Burkholderia multivorans* | BCEP7 | Patient isolate | [2] |
| *Burkholderia multivorans* | BCEP9 | Patient isolate | [2] |
| *Burkholderia pyrrocinia* | LMG14191^T^ | Soil | [3] |
|  |  |  |  |
| *Pseudomonas aeruginosa* | PA0882 | Patient isolate | This study |
|  | 1300-2 | Patient isolate | This study |
|  | 1315-3 | Patient isolate | This study |
|  | 1766-1 | Patient isolate | This study |
|  |  |  |  |
| *Escherichia coli* | EC231362 | Patient isolate | This study |
|  | EC294145 | Patient isolate | This study |
|  | EC236332 | Patient isolate | This study |

**References**:

1. Vandamme P, Henry D, Coenye T, Nzula S, Vancanneyt M, LiPuma JJ, Speert DP, Govan JR, Mahenthiralingam E: ***Burkholderia anthina* sp. nov. and *Burkholderia pyrrocinia*, two additional *Burkholderia cepacia* complex bacteria, may confound results of new molecular diagnostic tools**. *FEMS Immunol Med Microbiol*. 2002 **3**;33(2):143-9.
2. Ho CC, Lau CC, Martelli P, Chan SY, Tse CW, Wu AK, Yuen KY, Lau SK, Woo PC: **Novel pan-genomic analysis approach in target selection for multiplex PCR identification and detection of *Burkholderia pseudomallei*, *Burkholderia thailandensis*, and *Burkholderia cepacia* complex species: a proof-of-concept study.** *J Clin Microbiol* 2011, **49:**814-821.
3. Palleroni NJ, Holmes B. ***Pseudomonas cepacia* sp. nov., nom. rev.**. *Int. J. Syst. Bacteriol*. 1981 **31**: 479-481.
4. Vandamme P, Holmes B, Vancanneyt M, Coenye T, Hoste B, Coopman R, Revets H, Lauwers S, Gillis M, Kersters K, Govan JR: **Occurrence of multiple genomovars of *Burkholderia cepacia* in cystic fibrosis patients and proposal of *Burkholderia multivorans* sp. nov**. *Int J Syst Bacteriol*. 1997, **47**(4):1188-1200.
