# Supplementary material for: Metabolomic profiling of Burkholderia pseudomallei using UHPLC-ESI-Q-TOF-MS reveals specific biomarkers including 4-methyl-5-thiazoleethanol and unique thiamine degradation pathway
Source: Cell Biosci. 2015 Jun 2;5:26. doi: 10.1186/s13578-015-0018-x (PMC4475313; doi:10.1186/s13578-015-0018-x)
Supplement: Additional file 2: Table S2. — Primers used in this study. [file 13578_2015_18_MOESM2_ESM.doc]

**Additional file 2: Table S2** Primers used in this study

| Gene Targets | Primer Sequences | Purpose |
| --- | --- | --- |
| *thiM* | LPW27694 5′-GCCGCGAACCTGAGCGCG-3′  LPW27695 5′-GATCAGCGCGCCGAGCGC-3′ | Genomic DNA amplification of *B. pseudomallei* and *B. thailandensis* |
| *thiM* | LPW27790 5′-TACGACGCGATCGTGCAGGAACTG-3′  LPW27791 5′-ATGCCGTCCGTCACGTAGTCGATC-3′ | cDNA amplification of *B. thailandensis* |
| *thiI* | LPW29028 5′-GCGACCATCTTCCGATAGAG-3′  LPW29030 5′-CTCGACTTCAAGTTCCTG-3′ | Genomic DNA and cDNA amplification of *B. pseudomallei* and *B. thailandensis* |
| *apoB* | LPW27700 5′-GCTCGGCCAGAACATGCTGA-3′  LPW27701 5′-CGCAGCGACGTGTCCTTC-3′ | cDNA amplification of *B. pseudomallei* and *B. thailandensis* |
